# Role of Targeted Therapy in Dilated Cardiomyopathy: The Challenging Road Toward a Personalized Approach

**DOI:** 10.1161/JAHA.119.012514

**Published:** 2019-06-01

**Authors:** Job A. J. Verdonschot, Mark R. Hazebroek, James S. Ware, Sanjay K. Prasad, Stephane R. B. Heymans

**Affiliations:** ^1^ Department of Cardiology CARIM Maastricht University Medical Centre Maastricht The Netherlands; ^2^ Department of Clinical Genetics Maastricht University Medical Centre Maastricht The Netherlands; ^3^ Cardiovascular Research Centre Royal Brompton & Harefield Hospitals NHS Trust London United Kingdom; ^4^ National Heart and Lung Institute Imperial College London London United Kingdom; ^5^ London Institute of Medical Sciences Imperial College London London United Kingdom; ^6^ Netherlands Heart Institute Utrecht the Netherlands; ^7^ Department of Cardiovascular Research University of Leuven Belgium

**Keywords:** dilated cardiomyopathy, personalized, phenotyping, therapy, Cardiomyopathy, Genetics, Treatment

Dilated cardiomyopathy (DCM) is characterized by LV (left ventricular) or biventricular systolic dysfunction and dilation, not explained by abnormal loading conditions or coronary artery disease.^1^ DCM has a prevalence between 1 in 250 to 1 in 500. End‐stage DCM is the leading indication for heart transplantation. DCM is a condition of heterogeneous pathogenesis, emphasizing the importance of in‐depth geno‐ and phenotyping in this multifactorial disease. Using contemporary diagnostic workup, an underlying pathogenesis can be identified in between 50% and 75% of patients with multiple causes seen in ≈25%.^2^

Current therapy for DCM is mainly based on neurohumoral blockade as for other forms of HF (heart failure) with reduced ejection fraction.^3^ In spite of optimal medical therapy, the premature morbidity and mortality rate remains unacceptably high, driving the need for novel and more individualized therapeutic options. However, targeted therapy is largely lacking in clinical practice, but may be of significant added value as seen in the field of oncology.^4^ Herein, the development of targeted therapies has greatly improved outcome in many cancer patients. The recently proposed Morphology, Organ Involvement, Genetic, Etiology, Stage of disease nomenclature aims to better classify the multifactorial pathogenesis of DCM including upstream causes such as genetic variants and acquired diseases—toxic agents, viruses, immune disorders, hormonal changes, and arrhythmias—that interact and lead to overlapping disease‐driving molecular mechanisms that finally impair myocardial function.^1^, ^2^ These downstream disease‐driving mechanisms can contribute “variably” to the disease in the same patient. As such, parallels to oncology are challenging because biologic processes are not point mutations in a specific oncogene, but rather broad biologic pathways. Nevertheless, the Morphology, Organ Involvement, Genetic, Etiology, Stage of disease classification may provide the first step toward improved classification and a more personalized approached to care. Using imaging complemented by genetics, blood biomarkers, and occasionally cardiac sampling, a refined diagnosis of the underlying causes and downstream key processes can provide targeted treatment strategies. Furthermore, well‐characterized geno‐ and phenotyping provides further understanding of the individual prognosis and disease penetrance within a family.^2^ The extensive phenotyping of a patient should eventually lead to a diagnosis at the individual level, determining cause and downstream molecular process activity in the patient. Such “fingerprinting” of a patient could be the determinant for the treatment that will be most beneficial for the individual.

Many trials involving new therapies targeting key disease‐driving mechanisms were published in the past decade. Although results are often promising, almost none of them have yet emerged in the mainstream in guideline‐driven management of DCM patients. Still, many are promising, and the current overview aims to translate the information from these studies into opportunities for future novel or validation research.

## Pathophysiology

**Figure 1:**
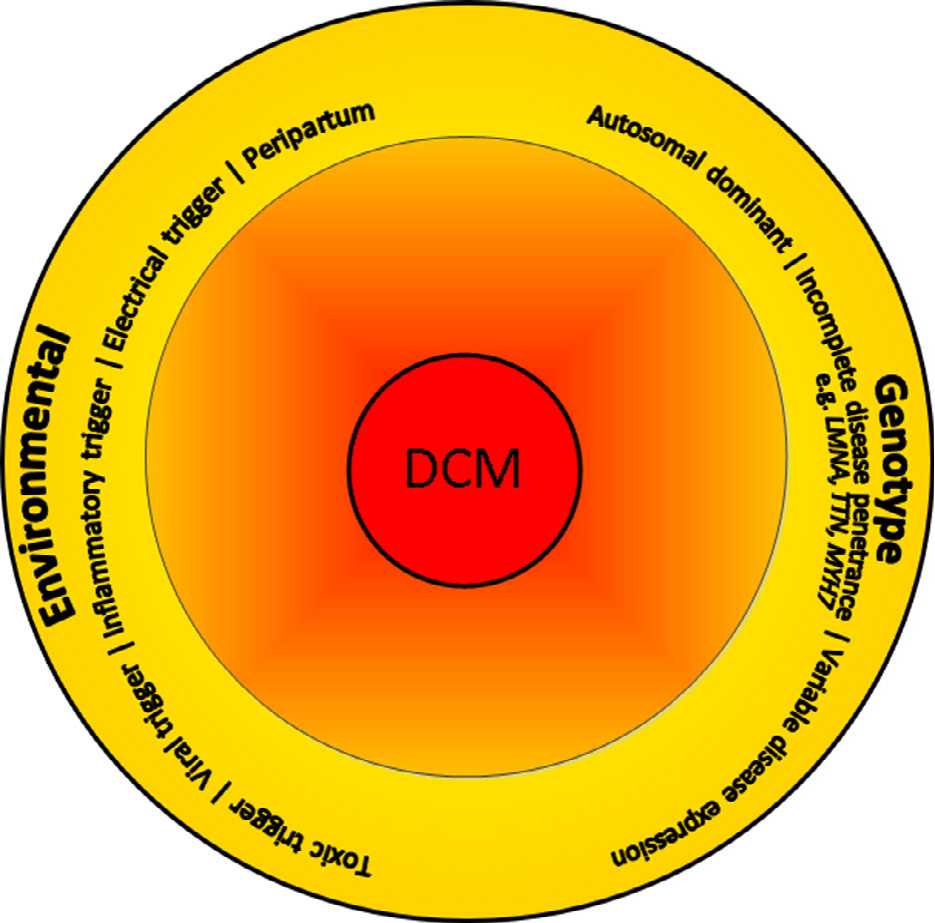
Different upstream environmental triggers on an individual genetic background can lead to dilated cardiomyopathy. DCM indicates dilated cardiomyopathy characterized by dilatation of the left and/or right ventricle and reduced cardiac function in the absence of ischemia and/or hemodynamic stress.

DCM is best regarded not a single disease entity, but rather as a nonspecific phenotype, with a final common response of the myocardium to a number of genetic and environmentally acquired insults, which were extensively discussed in a previous review.^1^ This in turn leads to variable presentation, and often patients do not have a clinically recognized acute onset of the cause of the disease. Therefore, knowledge regarding the exact stages and molecular changes for most causes is quite limited. Importantly, overall they share similar downstream pathophysiological mechanisms, which may have contributed “variably” in the same patient, ultimately leading to the end‐stage phenotype of cardiac dilation and systolic dysfunction (Figure 1). Thus, the finesse lies in understanding and recognizing the upstream pathogenesis or trigger to create a fingerprint of the key disease‐driving molecular mechanisms, so timely initiation of targeted therapy can be done to prevent irreversible progression of the disease. Several key molecular processes are at play during the development of DCM, including altered cardiac metabolism and fibrosis, which provide novel opportunities for treatment.

## Clinical Presentation

The majority of DCM patients will present when overt disease already has developed. Often the initial trigger or exact start of symptoms or “tipping point” is not known. Only a minority of patients will have symptoms at onset, largely depending on the cause, with peripartum or (viral) myocarditis being classic examples.^5^, ^6^ In the latter, the acute onset can range from days to weeks. Infection by a virus triggers inflammation and cardiac injury, and subsequently there is a shift to an adaptive immune response. Of note, in many subjects the damage is minimal and no DCM develops.^7^ Following acute myocarditis, a subset of ≈25% to 30% may deteriorate, with worsening HF culminating in end‐stage DCM. They remain at increased arrhythmic risk both in the short and long term.^7^ Whether this is also the case in other upstream causes of DCM is not known, because their acute pathological phases, if any, are difficult to recognize because of the slow onset of symptoms.

Because most DCM patients will present themselves when significant adverse remodeling is already manifest, focusing on the downstream molecular pathways at play throughout this phase is clinically most relevant. Importantly, several key processes may overlap during the disease course, which makes estimating disease duration challenging. Therefore, targeting key disease‐driving processes at the time of evaluation could be opportunities for treatment strategies, irrespective of the disease duration. Moreover, approximately one third of DCM patients with recovered LVEF (left ventricular ejection fraction) may relapse during the chronic disease.^8^, ^9^ Indeed, abrupt worsening of cardiac function or an increased ventricular arrhythmic burden can be driven not only by DCM progression but also by the development of new comorbidities, such as common cardiovascular risk factors.

## Precision Phenotyping

**Figure 2:**
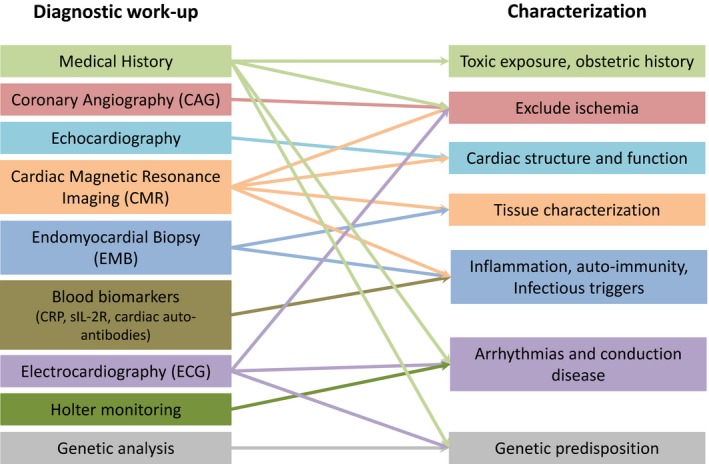
Complete diagnostic workup of a dilated cardiomyopathy patient to characterize the cardiac function and underlying cause. CRP indicates C‐reactive protein; sIL2‐R, soluble interleukin 2 receptor.

Phenotyping is essential for a heterogeneous disease such as DCM. A previous study in patients with HF with preserved ejection fraction elegantly demonstrated the benefits of deep phenotyping in combination with unbiased clustering analysis (so‐called phenomapping).^10^ Phenomapping in the heterogeneous HF with preserved ejection fraction population defined more homogeneous patient subclasses that benefit from specific therapies, showing the importance of structural phenotyping. Phenotyping in DCM is essential to provide optimal treatment, because the key targets for treatment per patient are dependent on the upstream cause and downstream active processes, reflecting patient subgroups (Table 1). Many tools can help us to determine the upstream trigger(s), which are extensively reviewed elsewhere (Figure 2).^1^ It is key to acquire a detailed history combined with clinical examination and echocardiography or CMR (cardiac magnetic resonance) assessment. Genetic testing and cardiac tissue sampling are useful in establishing underlying causes or susceptibilities. Detailed characterization of remodeling, particularly with CMR, provides further insight into cause and prognosis, and may guide treatment strategy. Specifically, it is useful to accurately quantify the degree of remodeling, to look for inflammation, exclude underlying infarction, and to assess the interstitial and replacement patterns of fibrosis.^11^, ^12^, ^13^ Moreover, genetic testing has evolved tremendously in the past few years from single‐gene to whole‐genome sequencing. The translation of this large generation of data toward clinical utility remains challenging because we often do not have enough knowledge to classify all variants found. Therefore, most centers performing genetic evaluations use a limited panel constituting ≈50 genes that are known to be DCM associated. Titin (*TTN*), lamin A/C (*LMNA*), and myosin heavy chain 7 (*MYH7*) are the most well‐known genes that are causative of DCM. Genetic analysis shows its importance as gene–environment interactions show prognostic value and determine the severity of disease presentation.^2^, ^14^ For example, alcoholic cardiomyopathy has a more severe disease presentation when there is an underlying *TTN* mutation.^14^ Truncating *TTN* variants are also seen in the general population, suggesting it does not always lead to a clear phenotype.^15^ Also, rodent studies show a very mild effect of *TTN* variants on cardiac function in the absence of an additional trigger.^15^, ^16^ However, *TTN* variants do often give a subclinical phenotype in the general population, probably predisposing to HF under stress conditions.^15^ The understanding of modifying environmental and/or genetic factors unveiling the cardiac phenotype is an important area of research. This highlights the importance of a complete workup in a patient, because 1 cause does not exclude others and often the underlying cause includes a second “insult” to unmask the phenotype. The goal lies in identification of those patients at risk of developing DCM and use of a combined diagnostic approach for earlier disease detection, with the opportunity to delay or possibly arrest disease progression.

**Table 1:** Diagnostic Options and Their Potential Treatment Targets of Specific DCM Causes

Causes and Processes	Diagnostic Workup	Positive Test Result	Targets for Treatment
Genetic	Detailed family history, genetic testing using gene panels (in adult disease) and WES (in pediatric disease), identifying specific clinical features (eg, early conduction disease in *LMNA*)	Finding of a variant that is classified as a pathogenic mutation with or without familial DCM	Early ICD therapy in *LMNA* DCM; p38 signaling in *LMNA* DCM; gene correction in patients with truncating variants
Toxic (alcohol/drug, cardiotoxic chemotherapy)	Detailed history of toxin exposure; possibility for urine toxicology screen	Disease onset during/after toxin exposure; regression or resolution after withholding	No specific targets: withholding or reducing exposure; use of cardioprotective agents in anthracycline toxicity (dexrazoxane)
Inflammation	EMB, blood sampling (eg, sIL2‐R, CRP, galectin‐3), CMR	Immune cell infiltration in EMB, increased T2‐signal, raised CRP/ESR	Pro‐inflammatory pathways (eg IL‐1β)
Autoimmune disease	EMB, blood sampling, imaging, autoantibody screen, presence of extracardiac features	Presence of inflammation and positive autoantibody titers	Auto‐antibodies (eg, β(1)‐AABs)
Viral	EMB	Cardiotropic virus presence with a viral load >500 copies/μg DNA	Virus and subsequent cardiac inflammation
Electrical	12‐lead ECG, ambulatory ECG monitoring	>10 000 to 25 000 PVCs/d; (supra)ventricular tachycardia	Abnormal electrical pathways
Peripartum	(history of) Pregnancy	Disease onset during pregnancy up to 6 mo postpartum	Cleaved 16 kDa N‐terminal fragment of prolactin
Cardiac metabolism	Blood, tissue and/or urine metabolomics	Elevated acylcarnitines, increase in ketone bodies	Multiple strategies possible interfering with the metabolic substrate switch (mainly involving mitochondrial pathways)
Cardiac fibrosis	EMB, CMR, blood	Increased CFV; midmyocardial LE; increased fibrosis blood markers	RAAS‐pathway; angiotensin II‐galectin‐3‐interleukin‐6 axis; matricellular proteins; syndecan‐4‐osteopontin‐lysyl oxidase‐like axis

AAB indicates autoantibodies; CFV, collagen fraction volume; CMR, cardiac magnetic resonance; CRP, C‐reactive protein; DCM, dilated cardiomyopathy; EMB, endomyocardial biopsy; ESR, erythrocyte sedimentation rate; ICD, implantable cardiac defibrillator; LE, late enhancement; PVC, premature ventricular complex; RAAS, renin‐angiotensin‐aldosterone system; WES, whole exome sequencing.

## Current State of Guideline‐Driven Therapies

The current treatment of DCM patients does not vary from general HF management, with the pharmacological cornerstone consisting of β‐blockers, RAS (renin‐angiotensin system) inhibitors, aldosterone antagonists, and diuretics.^17^ One of the most recent guideline‐changing breakthroughs in general HF treatment was the introduction of the angiotensin receptor‐neprilysin inhibitor sacubitril‐valsartan.^18^ The major PARADIGM (Prospective Comparison of ARNI with ACEI to Determine Impact on Global Mortality and Morbidity in Heart Failure) trial showed a risk reduction of death and HF hospitalization in chronic HF patients treated with sacubitril‐valsartan instead of enalapril. About 60% of the included patients had an ischemic cause, making the drug still relatively new in the field of DCM. The results of the PARADIGM trial have been analyzed post hoc in several subgroups.^19^ An opportunity remains to perform an additional post hoc analysis in only nonischemic HF patients to evaluate its effect in this subgroup.

## Targeting the Underlying Cause of DCM: Identifying Upstream Causes

In DCM it would be the goal to initiate therapies targeting key molecular processes driven by upstream causes. Knowledge regarding causes of DCM has expanded significantly over the past few years. The increasing number of patients in registries and combining international databases provides us with valuable information regarding clinical presentation and prognosis of specific causes. Current guidelines do not include treatment strategies directed at key molecular processes driven by upstream causes, because evidence is mostly coming from single‐center pilot and retrospective cohort studies (Table 2). Now is the time to make the next step toward multicenter, randomized trials (Table 3).

**Table 2:** Overview of All Studies Investigating Cause‐Directed Treatments in Dilated Cardiomyopathy Patients

	Therapy	Year	Brief Study Design	Nr	Follow‐Up	Outcome	References
Genetics							
Signal transduction alteration	ARRY‐371797	2018	RCT	Estimation 160	6 mo	Study is still ongoing, estimated study completion 2020	NCT03439514
Acquired triggers							
Inflammation	Prednisone+Azathioprine	2001	RCT	84	24 mo	C	20
	Prednisone+Azathioprine	2009	RCT	85	6 mo	C	21
	Prednisone+Azathioprine	2018	Retrospective, case–control matched study	180	31 mo	C, L	22
	Anakinra	2016	RCT	30	14 d	Decreased systemic inflammation	23
	Anakinra	2017	RCT	60	24 wks	S	24
Viral	Interferon‐β	2003	Open	22	24 wks	C	25
	Intravenous Immunoglobulins	2010	Open	17	6 mo	C	26
	Intravenous Immunoglobulins	2018	RCT	50	6 mo	Study recently completed, results are pending	NCT00892112
Auto‐immunity	Immunoadsorption and Ig replacement	1996	Open	8	Variable	C, S	27
	Immunoadsorption	2000	Case–control study, adsorption in addition to OMT	34	12 mo	C, S	28
Toxic	Mesenchymal stem cells	2018	RCT	Estimation 36	12 mo	Study is still ongoing, estimated study completion 2019	NCT02509156
Peripartum	Bromocriptine	2010	Open, randomized pilot study, Br on top of OMT	20	6 mo	C, L	29
	Bromocriptine	2017	RCT	63	6 mo	Short‐ and long‐term Br use in addition to OMT are equally beneficial	30
Electrical	Cathether ablation of AF	2004	Prospective, case–control matched study	58	12 mo	C, S	31
	Cathether ablation of PVC	2011	Retrospective cohort study	69	11 mo	C	32
	Catheter ablation vs antiarrhythmic drugs	2014	Retrospective case–control matched study	510	12 mo	C, S	33
	Rhythm vs rate control	2009	RCT	61	12 mo	C, S	34
	Rhythm vs rate control	2010	RCT	614	36 mo	Lenient rate control is as effective as strict rate control and is easier to achieve	35
	Catheter ablation vs amiodarone	2016	RCT	203	24 mo	Superior in achieving freedom from AF, better long‐term outcome	36
Pathophysiological processes							
Myocardial damage	Bone marrow–derived stem cells	2015	RCT	60	12 mo	C	37
	Hematopoietic stem cells	2013	Open, randomized	110	60 mo	C, S, L	38
	Mesenchymal stem cells	2017	RCT	37	12 mo	C, S	39
Biomechanical defects	MYK‐491	2018	RCT	Estimation 56	49 d	Study is still ongoing, estimated study completion 2019	NCT03447990
Altered cardiac metabolism	Etomoxir	2000	First trial with etomoxir in DCM individuals	9	3 mo	C	40
	Perhexiline	2015	RCT	50	2 mo	S	41
	Trimetazidine	2008	RCT	19	3 mo	C	42
	Trimetazidine	2013	RCT	80	6 mo	C, S	43
Fibrosis	Spironolactone	2005	Open	25	12 mo	C	44

AF indicates atrial fibrillation; C, increased cardiac function; DCM, dilated cardiomyopathy; L, better long‐term survival; NA, not applicable; OMT, optimal medical therapy; RCT, randomized controlled trial; S, decreased symptoms and improved functional status.

**Table 3:** Current Situation and Future Directions for Targeted Therapies in Dilated Cardiomyopathy

	Current State	Ongoing Projects	Future Directions
Upstream triggers			
Genetic	Prevention with device therapy in gene mutations susceptible to malignant arrhythmias (ie, *LMNA*)	Phase 3 clinical trial using p38 inhibition in *LMNA* DCM patients	Unravel molecular consequences of specific gene mutations Gene correction therapies towards a clinical application
Inflammation	No guideline‐directed therapy; although there is evidence from retrospective studies showing benefit from immunosuppression	Phase 2B RCT in acute myocarditis using anakinra vs standard care	Multicenter RCT using immunosuppressive therapy in inflammatory DCM
Auto‐immunity	No guideline‐directed therapy; although there is evidence showing benefit from immunoadsorption	···	Multicenter RCT using immunoadsorption in DCM with cardiotoxic autoantibodies
Viral	No guideline‐directed therapy; although there are retrospective studies and case reports showing benefit from IVIg	Phase 3 RCT using IVIg for chronic PVB19‐related DCM	RCT using specific antiviral therapies (val/‐ganciclovir) Multicenter RCT for IVIg if phase 3 trial is positive
Toxic	No guideline‐directed therapy; withholding or reducing exposure has been shown to be the most effective; in some cases, cardioprotective compounds for anthracycline toxicity are advised.	Phase 1 RCT using MPCs in anthracycline‐induced DCM	Unravel molecular changes in cocaine‐induced DCM Phase 2 RCT using stem cell therapy in cardiotoxic chemotherapy‐induced DCM Define the timing, dose, and duration of prophylactic therapy to prevent HF onset in those patients receiving cardiotoxic chemotherapy at risk
Electrical	Early treatment of electrical disturbance (AF, ablation; left bundle branch block, CRTD)	···	Better insight in the pathomechanisms of the interplay between HF and electrical disturbances to detect their causal relationships and better stratify patients who will benefit from (device) therapy
Peripartum	No guideline‐directed therapy; although 1 prospective study showed benefit from bromocriptine	···	A placebo‐controlled study with bromocriptine to assess safety and efficacy More data regarding long‐term outcome of PPCM after bromocriptine use
Downstream processes			
Biomechanical defects	No guideline‐directed therapy	Phase 1 and phase 2 RCT using MYK‐491 in DCM	Follow‐up research based on results of current phase 1 and 2 RCT Investigate the role of omecamtiv in (genetic) DCM
Myocardial damage	No guideline‐directed therapy; although multiple studies investigated the benefit from stem cell therapy	···	Phase 3 RCT combining G‐CSF and BMC in DCM as follow‐up on the REGENERATE‐DCM
Cardiac metabolism	No guideline‐directed therapy; although multiple compounds have been investigated showing benefit	···	More data regarding long‐term outcome of DCM after trimetazidine Investigating extracardiac effects of perhexiline use
Cardiac fibrosis	No guideline‐directed therapy	RCT using ICD or ILR implantation in DCM patients with LGE on CMR	Specific antifibrotic medication tailored to arrhythmic burden or reversibility of fibrosis Exploring the role of novel antifibrotic therapies with regard to cardiac fibrosis (ie, vanticumab, simtuzumab)

AF indicates atrial fibrillation; BMC, bone marrow‐derived stem cells; CMR, cardiovascular magnetic resonance; CRTD, cardiac resynchronization therapy device; DCM, dilated cardiomyopathy; G‐CSF, granulocyte colony‐stimulating factor; HF, heart failure; ICD, implantable cardioverter‐defibrillator; ILR, implantable loop recorder; IVIg, intravenous immunoglobulin; LGE, late gadolinium enhancement; MPC, mesenchymal progenitor cells; PPCM, peripartum cardiomyopathy; PVB19, parvovirus B19; RCT, randomized controlled trial.

### Genetic Causes

Inheritance of a pathogenic gene variant is currently considered an irreversible cause and clinical management is based on the increasing genotype–phenotype knowledge. To date, the monogenetic dogma is applied in clinical practice, whereas genetic DCM is mainly considered an autosomal dominant disease in which 1 gene variant contributes to the DCM phenotype. However, increasing insights suggest that DCM, besides being a monogenic disease, is potentially a polygenic, complex disease involving the interaction among multiple gene variants. Whether this complex disease model will result in therapy guidance is yet to be discovered. Currently, guided therapy in the field of (mono)genetics can roughly be divided into 3 different approaches. The first approach is based on *amelioration of the clinical consequences* related to the specific gene mutation, based on current genotype–phenotype knowledge. The best example is a lower threshold for cardiac defibrillator implantation in patients with a pathogenic lamin A/C (*LMNA*) mutation, which is related to a higher risk of rhythm disturbances and sudden cardiac death.^45^ For most other mutations, there is still a dearth of clinical and prognostic data to understand clinical consequences. Also, they often have a lower disease penetrance and more clinical variability among gene mutation carriers compared with *LMNA* mutations.^46^ This is likely because of the effect of additional environmental factors needed to trigger the phenotype on top of the genotype, creating the concept of susceptibility genes.^2^, ^6^, ^14^ This is especially relevant for truncating *TTN* mutations, which are the most prevalent among genetic causes for DCM.

A second approach is to *target the molecular consequences* of a specific gene mutation. Most cardiomyopathy‐related genes and their subsequent proteins have specific functions in the cardiomyocyte. Loss (or gain) of function of some of these proteins will lead to intracellular changes of the signal transduction for which the cardiomyocyte tries to adapt. These molecular changes have been well investigated in *LMNA*‐mutated mice.^47^ The most distinct change is the increased cardiac activity of the ERK1/2, JNK, and p38 MAP kinases. Interestingly, when the mice were treated with the p38 inhibitor ARRY‐371797, it prevented LV dilation and deterioration of EF.^47^ In addition, p38 inhibition was able to rescue *LMNA*‐related severe biomechanical defects in neonatal rat ventricular myocytes.^48^ The increasing evidence of beneficial p38 inhibition led to an international phase 3 clinical trial, which is currently running (NCT03439514). This double‐blinded, randomized, placebo‐controlled study investigates the benefit of ARRY‐371797 on change in 6‐minute walk distance at 12 weeks in 160 patients with symptomatic DCM caused by a *LMNA* gene mutation, which will be the first study involving genotype‐specific therapy. With the increasing possibilities of transcriptomics and proteomics, it is likely that the altered signal transduction of other gene mutations will be unraveled. Truncating *TTN* mutations are a good example, as evidence is accumulating regarding changes in the cardiac metabolism and energy homeostasis.^49^ The increase in the energy metabolism could be a mechanism of metabolic compensation for the sarcomere insufficiency.^50^ This change in metabolism related to truncating *TTN* mutations can be a valid downstream target for intervention to prevent disease onset toward DCM.

The ultimate therapy is to *address the genetic perturbation* in the individual patient. Several methods are under investigation to achieve this therapeutic goal such as (1) CRISPR/Cas9, which can target specific single mutations; (2) exon skipping, targeting all mutations present in 1 or 2 exons and their associated introns; and (3) gene replacement targeting all mutations at once by gene transfer of the full‐length cDNA.^51^ Since its discovery in 2013, CRISPR/Cas9 is now becoming a valuable tool to investigate pathogenicity of gene mutations, create experimental models, and to develop a genome‐editing therapy for specific gene mutations such as *DMD*.^52^, ^53^ In vivo genome editing with CRISPR/Cas9 restored dystrophic cardiomyopathy structurally and functionally in an animal model.^52^ Exon skipping is especially useful in truncating mutations, as the exon containing the new stop‐codon can be removed, preventing an incomplete transcript. Truncating mutations in *TTN* are the most well known, and exon skipping in patient‐specific cardiomyocytes derived from induced pluripotent stem cells could be rescued, preventing defective myofibril assembly and stability.^54^ Also, exon skipping prevented the development of DCM and improved contractile performance in *TTN*‐mutated mice.^54^ Gene replacement is a promising therapy for specific severe forms of genetic DCM when a mutation results in a low level or absence of the corresponding protein, which is mostly seen in pediatric cases of DCM. Evidence for the feasibility of gene replacement in a mouse model and human induced pluripotent stem cell–derived cardiomyocytes carrying gene mutations in *MYBPC3* has been shown.^51^ Gene replacement has been realized by transducing the cells with AAV (adeno‐associated virus).^55^ This successfully increased the expression of *MYBPC3* transcripts and suppressed the disease phenotype. Although most of the genome editing has been shown to be feasible and promising in cells and mice, there are still many questions that need to be answered before progressing to human trials with the 3 techniques mentioned. These include the safety of viral delivery and delivering the vector to the right place in the right dose, which will be challenging. Gene replacement therapy depends on AAV with a high cardiac tropism and a cardiac‐specific promotor, which is AAV9 in mouse models. However, the best AAV serotype for the human heart still needs to be determined. Nonspecific cardiac tropism can lead to off‐target effects, strong host immunogenicity against the virus, insufficient AAV potency, and efficacy and incomplete incorporation.^56^ Interestingly, intracoronary infusion of AAV1‐SERCA2a in 123 patients with HF with reduced ejection fraction was not associated with improvement in clinical outcome.^57^ Despite the failure of this study, these findings should stimulate further research into the use of gene therapy to treat HF patients.

### Inflammatory Triggers

So far, 3 randomized trials have examined the effect of immunosuppressive therapies in DCM.^20^, ^21^, ^58^ The 2 largest of these trials assessed the effect of prednisone and azathioprine. However, they were conducted before the era of quantitative immunohistochemistry for the assessment of infiltrates, using solely hematoxylin and eosin staining and the Dallas criteria.^20^, ^58^, ^59^ In addition, molecular analysis for viral presence in myocardial biopsies was not available. Both trials showed no beneficial effect on mortality, where the IMAC (Intervention in Myocarditis and Acute Cardiomyopathy) trial also failed to show beneficial effects on cardiac function. Interestingly, Frustaci et al demonstrated improved and sustained cardiac function in DCM patients treated with immunosuppressive therapy in addition to HF therapy as compared with those who received standard HF treatment alone. The importance of discrimination between virus‐positive and ‐negative patients was exemplified by a post hoc stratification of patients treated with prednisone and azathioprine, showing beneficial effects predominantly in the virus‐negative group.^60^ Subsequently, a prospective study was done using immunosuppressive therapy in 85 patients with aggressive cardiac inflammation, but the absence of virus revealed a significantly improved cardiac function at 6 months in the immunosuppression group as compared with standard HF therapy only. Besides the potential beneficial effects on cardiac function, long‐term transplantation‐free survival can also be improved using this immunosuppressive regimen.^22^ Based on these results, the most recent European Society of Cardiology recommendations state that immunosuppression may be considered in infection‐negative myocarditis refractory to standard therapy in patients with no contraindications to immunosuppression.^61^, ^62^ In contrast, these recommendations are not advocated by the American Heart Association guidelines.^63^ To definitively prove potential benefits of immunosuppression in this subset of patients, a multicenter trial was initiated but terminated because of a low inclusion rate (NCT01877746). Therefore, confirmation of a randomized, prospective, placebo‐controlled multicenter trial will be necessary before a consensus is reached in guidelines.

Recently, the role of IL (interleukin)‐1β blockade, namely, anakinra, has raised interest because clinical pilot studies in inflammatory DCM showed beneficial effects on hemodynamics, inflammation, and clinical performance.^23^, ^24^ Anakinra is a nonglycosylated protein that differs from the sequence of the native IL‐1 receptor antagonist by 1 methionine added to its N‐terminus. The IL‐1 family of ligands and receptors is the main cytokine family associated with acute and chronic inflammation. Recently, 30 patients with acute decompensated HF and elevated C‐reactive protein levels were randomized to either receive anakinra or matching placebo.^23^ Anakinra reduced C‐reactive protein by 61% versus baseline, compared with a 6% reduction among patients receiving placebo. Moreover, anakinra was associated with a greater recovery in LVEF compared with placebo. No significant differences between treatment groups in the initial length of stay or total hospital days during the 14 days were observed. Based on these favorable results, safety and efficacy of anakinra was evaluated in the REDHART Trial (Recently Decompensated Heart Failure Anakinra Response Trial), involving 60 patients with recently decompensated heart failure and systolic dysfunction. Anakinra treatment reduces serum C‐reactive protein levels in a sustained manner and improves peak VO_2_. However, IL‐1B blockade does not improve cardiac function or improve prognosis in terms of death or HF hospitalization at 24 weeks as compared with placebo.^24^ However, the study was not powered to detect differences in outcome and should be used as an estimate of effect size to design appropriately powered studies. Moreover, the ACTION Study Group in France initiated a Phase 2B randomized controlled trial in acute myocarditis patients using anakinra versus standard care (NCT03018834). The study is estimated to be completed in 2021. Blockade of IL‐1B seems to be a promising therapy target in HF, as a recent subgroup analysis using placebo‐controlled data of the CANTOS (Canakinumab Anti‐Inflammatory Thrombosis Outcome Study) trial in which canakinumab (a monoclonal antibody against IL‐1B) showed a dose‐dependent reduction in HF hospitalization and HF‐related mortality in 2173 patients with HF at baseline.^64^ Hence, 1 of the inclusion criteria for the CANTOS trial was a baseline C‐reactive protein ≥2 mg/L, thus creating a selected population with signs of elevated systemic inflammation who benefitted from canakinumab treatment.

### Autoimmunity

The concept of immunoadsorption involves the removal of cardiotoxic autoantibodies, together with cytokines from the circulatory system, because they might cause damage to the myocardium.^27^, ^65^ To avoid infection, the adsorbed immunoglobulins (namely, IgG, IgA, and IgM) are replaced using administration of 0.5 g/kg body weight polyclonal IgG after treatment. Although studies are relatively small, an improvement of LV function is reported in ≈60% of patients treated.^28^ Using gene expression analysis from the myocardial tissue, responders and nonresponders can be potentially distinguished before treatment.^66^ Although these results seem attractive, confirmation by a randomized treatment trial is needed.

### Viral Triggers

These include antiviral therapies targeting cardiotropic viruses such as herpes simplex virus 1 and 2, cytomegalovirus, Epstein–Barr virus, varicella virus, human herpes virus 6 and 7, parvovirus B19, respiratory syncytial virus, hepatitis C virus, and HIV. Specific antiviral therapies comprise val‐/ganciclovir, acyclovir, foscarnet, high‐dose intravenous immunoglobulins, and antiretroviral therapy administration in HIV infection.^67^ Of note, only a few are tested in a trial setting for DCM, with limited randomized control data.

In enterovirus‐positive DCM patients, spontaneous enterovirus elimination and elimination using interferon‐β are both associated with clinical as well as hemodynamic improvement.^25^ Of note, half of these patients spontaneously eliminated their enterovirus without specific treatment, suggesting that not merely the presence of viral genomes but also the replication status and viral load are important criteria for antiviral therapy.^5^

Retrospective studies demonstrated that DCM patients with a high viral load may benefit from intravenous immunoglobulins.^26^ Although a randomized trial in 62 patients with recent‐onset DCM (<6 months) did not show beneficial effects on mortality and LVEF after 6 and 12 months, it lacked evaluation of viral infection and only 12% exhibited cardiac inflammation.^58^ Currently, a single‐center randomized controlled trial using high‐dose intravenous immunoglobulins (2.0 g/kg body weight) for chronic parvovirus B19–related DCM to reduce parvovirus B19 viral load was completed in August 2018, enrolling a total of 50 patients. (NCT00892112).

### Toxic Triggers

Toxic triggers are most known for structural and functional changes in the myocardium because of increased myocyte loss, which is largely irreversible.^68^, ^69^, ^70^ The main advice remains to reduce or withdraw the introduced toxic trigger such as excess alcohol, cocaine, or cancer therapy.

Anthracyclines are best known for their cardiotoxic effect. They manifest their cardiotoxicity via multiple mechanisms: increase in oxidative stress, modulation in topoisomerase activity, alteration in the multidrug‐resistant efflux proteins, and a decrease in mesenchymal progenitor cells.^70^ The decrease in mesenchymal progenitor cells reduces the cardioreparative capacity of the heart when it is exposed to stress.^71^ A phase I randomized, placebo‐controlled trial currently evaluates the safety and feasibility of administering mesenchymal progenitor cells to patients with anthracycline‐induced DCM (NCT02509156). This trial will lay the foundation for a specific treatment regarding chemotherapy‐induced DCM in addition to prompt treatment with general HF therapy.^72^ Multiple trials investigated the potential of prophylactic cardioprotective therapy using general HF medication or specific compounds such as dexrazoxane, an iron chelator that decreases the formation of superoxide radicals.^73^ However, current data do not support the routine prophylactic use of HF treatment in patients receiving cardiotoxic chemotherapies. In contrast, the use of dexrazoxane is licensed for clinical use in cancer patients undergoing anthracycline dosing in excess of 300 mg/m^2^ or epirubicin >550 mg/m^2^. Therefore, it may be considered in patients receiving these high doses of cardiotoxic chemotherapy.^74^ Ongoing studies will help to better define the timing, dose, and duration of prophylactic therapy to prevent HF onset in those patients at risk. Also, high‐throughput screenings to identify novel cardioprotective compounds are ongoing, although none of them reached clinical trials yet.^75^

A cocaine use disorder can lead to a broad spectrum of cardiovascular complications.^69^ Careful diagnostics are therefore important when seeing a patient with suspicion of cocaine‐related DCM. Cocaine exerts its toxic effect via multiple pathways: myocardial scarring, impaired intracellular calcium handling, apoptosis, increase of oxidative stress, and acute effects of the catecholamines. However, current treatment advice is to treat according to the general HF guidelines, and trials investigating specific treatment regimens are lacking.

Alcohol in low concentrations is beneficial for the heart, in contrast to the other toxic triggers.^68^ Long‐term heavy alcohol consumption, however, may lead to alcoholic DCM, most likely via structural damage to the cardiomyocyte leading to apoptosis.^68^ Also, ethanol and its metabolites are thought to be toxic for the sarcoplasm and mitochondria, altering calcium sensitivity at the myofilament level. Specific guidelines for the treatment of alcoholic DCM are lacking. Most studies focus on the clinical effect of alcohol decrease or complete abstention.^68^ Patients who reduced their alcohol intake to moderate levels had a similar survival and cardiac function recovery compared with complete alcohol abstainers.^76^ Overall, the alcoholic DCM patients had a better survival compared with the general group of DCM patients. Therefore, alcohol reduction in addition to general HF treatment is the keystone of therapy in alcoholic DCM.

### Electrical Triggers

Arrhythmia‐induced DCM is a well‐known potentially reversible condition in which DCM is induced or mediated by atrial or ventricular arrhythmias and has been extensively reviewed previously.^77^ It may follow many types of cardiac arrhythmia: supraventricular tachyarrhythmias, ventricular tachycardia, or frequent ventricular ectopy. However, the diagnosis of arrhythmia‐induced cardiomyopathy is often difficult, as both are frequently diagnosed simultaneously. DCM in response to an arrhythmia may take months to years to develop, although rapid declines in ventricular function with development of HF symptoms are also observed in recurrent (tachy)arrhythmias. If this beneficial response remains absent, one must consider other factors influencing the arrhythmic phenotype such as genetic mutations (ie, LMNA or SCN5A mutations).^45^ To date, the exact mechanisms of most genetic and nongenetic arrhythmias in DCM are not completely understood, but involve the loss of normal extracellular matrix and contractile dysfunction, alterations in cellular growth and viability, defects in Ca^2+^ handling, and neurohormonal activation results in DCM.^77^

Managing patients with suspected arrhythmic‐induced DCM involves attempting careful and aggressive control of rate and rhythm, with the focus on arrhythmia elimination by catheter ablation whenever possible. Identifying the underlying condition is important to predict treatment response. In a study of 27 patients with frequent PVCs (premature ventricular complexes) and DCM, 22 had improvement in LVEF following PVC suppression; 5 did not. Four of the 5 patients with LVEF that did not improve had evidence of irreversible myocardial fibrosis detected by late gadolinium enhancement CMR.^78^ A high PVC burden of >10 000 PVCs/d or >10% to 24% of total heartbeats/d can cause DCM. Reducing the PVC burden to <5000/d can improve LVEF.^32^ Moreover, elimination of PVCs with ablation has been shown to improve LVEF, ventricular dimensions, mitral regurgitation, and functional status. In an observational series, ablation was superior to antiarrhythmic therapy in reducing PVCs and improving LVEF.^79^

In AF (atrial fibrillation), several trials evaluating rhythm control compared with rate control strategies have been conducted. Although they did not focus on arrhythmic‐induced DCM, several lessons can be learned. In general, rhythm control does not provide a benefit in all‐cause mortality or worsening HF as compared with rate control, with similar findings in strict versus lenient rate control.^35^ In AF‐mediated DCM, rhythm control is superior to rate control in improving LVEF, pro‐brain natriuretic peptide levels, and quality of life.^34^ Moreover, in AF‐mediated DCM, restoring and maintaining sinus rhythm can accelerate clinical recovery and reverse DCM over several months.^33^ Indeed, restoration and maintenance of sinus rhythm by catheter ablation in patients with congestive HF and AF significantly improves cardiac function, symptoms, exercise capacity, and quality of life.^31^ In atrial flutter, catheter ablation is recommended in arrhythmia‐induced DCM, because rate control is more difficult than in AF.^36^

### Peripartum Cardiomyopathy

Peripartum cardiomyopathy is a form of DCM with deterioration of cardiac function typically between the last trimester of pregnancy and up to 6 months postpartum. It is recognized as a major cause of pregnancy‐related HF.^80^ To date, no evidence‐based specific therapy is recommended in the current guidelines. The exact disease pathophysiology is not known; however, high levels of cleaved 16‐kDa N‐terminal fragment of prolactin have been shown to be an important mediator. Bromocriptine is a dopamine‐D2‐receptor agonist and inhibits prolactin release. A pilot study in acute severe peripartum cardiomyopathy showed improvement in LVEF and prognosis when bromocriptine was added to standard HF therapy.^29^ A later randomized multicenter clinical trial confirmed these findings and showed no significant difference between short‐ and long‐term treatment with bromocriptine.^30^ Importantly, the trial did not include a nonuse control group, leaving many unanswered questions. Ongoing observatory registries will provide further information regarding benefit and follow‐up of bromocriptine use.^81^ Despite European experience and guidelines, a placebo‐controlled trial of bromocriptine for peripartum cardiomyopathy is very much needed.

## Targeting Downstream Molecular Processes Driving Disease Progression

Despite many downstream processes driving the progression to DCM overlap among different upstream causes, the timing and severity of these processes may vary. Clinical outcome is eventually determined by the interplay between trigger, host response, and therapeutic intervention. The alterations in cardiac metabolism and fibrosis are popular targets for intervention. In contrast, cardiomyocyte apoptosis, stretch, and damage are less suitable for intervention because they are often irreversible. However, cell therapy does provide potential as a future therapy to remedy cardiomyocyte damage and loss.

### Cardiomyocyte Dysfunction

DCM is characterized by biomechanical defects leading to inadequate cardiac contraction. Positive inotropic agents, such as dobutamine and milrinone, are indicated as a therapy in patients with end‐stage HF and cardiogenic shock.^82^ Milrinone is a bipyridine and inhibits phosphodiesterase‐3, eventually leading to an increased cellular calcium influx with subsequent stimulation of myocardial contractility. The OPTIME‐CHF (Outcomes of a Prospective Trial of Intravenous Milrinone for Exacerbations of Chronic Heart Failure) study was a large randomized trial to investigate the incremental value of milrinone in addition to standard HF therapy in patients with acute HF.^83^ This study showed significant side effects of milrinone, such as sustained hypotension and arrhythmias. A post hoc analysis showed that milrinone was associated with higher mortality and rehospitalization in ischemic HF but was neutral to beneficial in nonischemic HF patients.^84^ However, we should be careful in the interpretation of this post hoc analysis because the study was not initially designed for this purpose. For example, a subset analysis of the PRAISE (Propective Randomized Amlodipine Survival Evaluation) I study showed a significant reduction in mortality in the nonischemic subgroup treated with amlodipine.^85^ The well‐designed PRAISE 2 study, whose purpose was to investigate the potential of amlodipine to reduce mortality in patients with nonischemic HF, failed to show this effect with trends in favor of placebo.^86^ There are currently no therapies that address the underlying biomechanical causes in DCM; one of the newest therapies aims at increasing the contractile function of the heart with minimal adverse effects on myocardial relaxation. The compound, named MYK‐491, is currently being investigated in a randomized, double‐blind, placebo‐controlled, crossover single‐ascending dose phase 1b trial including DCM patients (NCT03447990). In contrast to milrinone, MYK‐491 is an allosteric activator of myosin, thereby not increasing calcium levels in the cardiomyocytes and limiting potential side effects. If the safety and tolerability and echocardiographic measures of cardiac contractility are found to be positive, MYK‐491 will be pursued in future research. A different inotropic compound is omecamtiv mecarbil, which showed favorable hemodynamic results in patients with chronic HF in a phase II clinical trial.^87^ In patients with acute HF, omecamtiv mecarbil did not meet the primary end point of dyspnea improvement.^88^ Both phase II trials (ATOMIC‐AHF [Acute Treatment with Omecamtiv Mecarbil to Increase Contractility in Acute Heart Failure] and COSMIC‐HF [Chronic Oral Study of Myosin activation to Increase Contractility in Heart Failure]) showed small increases in plasma troponin concentrations in treated patients. Overall, there was no relationship with omecamtiv concentrations and occurrence of adverse events. The origin of these raised troponin concentrations is unknown and needs to be addressed in larger outcome trials. A phase III morbidity/mortality trial in patients with chronic HF (NCT02929329) is currently recruiting patients. Interestingly, omecamtiv mecarbil was demonstrated to augment cardiac contractility in DCM using a DCM mouse model containing a tropomyosin mutation.^89^ This makes omecamtiv mecarbil a promising compound to treat DCM patients with sarcomeric gene mutations.

### Cardiomyocyte Damage and Loss

Stem cell therapy to compensate for damaged/lost myocardial tissue aims to improve cardiac function in HF.^39^ Although the main focus of this approach was on ischemic HF, multiple clinical trials investigated the value of cell therapy in DCM, which is extensively reviewed elsewhere.^90^ The most widely used method for delivery is intracoronary. Although shown to be safe and simple, these stem cells cannot reach the inadequately perfused myocardium. Also, homing and retention of the stem cells at the right location is still difficult. A variety of stem cell types are investigated in DCM: bone marrow–derived, hematopoietic, and mesenchymal stem cells. Initially, cytokines and stem cells were investigated separately and both had shown beneficial effects on intermediate outcome such as cardiac function.^38^, ^91^ REGENERATE‐DCM was the first phase II randomized, placebo‐controlled trial showing a significant improvement in cardiac function, symptoms, and biochemical parameters after a combined treatment of granulocyte colony‐stimulating factor and bone marrow–derived stem cell therapy.^37^ The results of the REGENERATE‐DCM cannot be translated to long‐term outcome; therefore a phase III trial is necessary to investigate these promising beneficial effects of combining granulocyte colony‐stimulating factor and bone marrow–derived stem cells in DCM.

### Altered Cardiac Metabolism

One of the key hallmarks in HF is the decreased oxidative metabolism of the cardiomyocyte.^92^ This in turn leads to a switch from fatty acid oxidation toward increased glucose metabolism to fulfill the energy demand of the contracting cardiac muscle. A large part of the glucose utilization is used for anaerobic glycolysis, which is an energy‐inefficient process. Eventually, the accumulating metabolic changes in HF will lead to an energy deficit, which maintains the progression of the disease.^92^ Multiple compounds have been tested to intervene in different stages of metabolic changes, as extensively described in a previous review.^92^

One possible intervention mechanism is to stimulate the heart to use glucose as primary substrate by inhibiting the fatty acid oxidation. In this case, the heart will use its available oxygen in a more efficient way. Trimetazidine is a thiolase I inhibitor. Thiolase I catalyses the last step of the beta‐oxidation in the mitochondria and its inhibition will shift the substrate utilization toward glucose. Use of trimetazidine in addition to optimal medical treatment showed a significant increase in LVEF in DCM.^42^ These findings were replicated in a later double‐blinded study including DCM patients with diabetes mellitus.^43^ In addition, they also showed that the inflammatory response was decreased, and the physical performance of the patients was better. A recent meta‐analysis of trimetazidine use in DCM showed its effectiveness regarding LVEF improvement and reduction in cardiac dimensions.^93^ Although there is no long‐term follow‐up in DCM patients, a meta‐analysis showed a decreased risk for hospitalization but no effect on all‐cause mortality in general HF.^94^ Trimetazidine is the compound for metabolic support that is the most ready for larger‐scale trials in DCM.

Carnitine palmitoyl transferase is part of the acyl‐carnitine shuttle of the mitochondria, which plays a crucial role in fatty acid transport. Etomoxir is a compound that inhibits carnitine palmitoyl transferase and thereby inhibits the beta‐oxidation, stimulating glucose as the primary substrate.^92^ The first trial with etomoxir included 90% nonischemic DCM patients and showed improvements in LVEF and clinical status.^40^ However, a larger follow‐up study with a general HF population was terminated prematurely because of adverse side effects.^95^ Perhexiline is an alternative carnitine palmitoyl transferase inhibitor that showed its value in hypertrophic cardiomyopathy and ischemic HF.^96^ Recently, a phase 2 randomized, double‐blinded, placebo‐controlled study was published, only including nonischemic DCM patients.^41^ Although the study noted no benefit in cardiac function, the symptoms improved in patients receiving perhexiline. More importantly, treatment with perhexiline increased the energy status of the heart without detectable changes of substrate utilization. This suggested that the treatment duration in this study was too short or that the effects of perhexiline are much broader than only carnitine palmitoyl transferase inhibition. However, perhexiline was taken off the market in many countries because of its toxicity in patients with CYP2D6 polymorphisms, which constitutes a significant proportion in the white population.

Although metabolic changes are a major effect of HF in the heart, therapy targeting these changes has not seen clinical implementation yet, partly because of our incomplete knowledge regarding the specific metabolic changes in the heart. New methods such as metabolomics will shed more light on the molecular changes, which will help us to better identify the best patients and stage of the disease when metabolic support can be the most effective. The current available larger studies used heterogeneous HF populations, probably leading to the mixed results. Preclinical and clinical data in the general HF population with newer molecules were promising,^92^ and investigating these newer compounds in DCM would be valuable.

### Cardiac Fibrosis

Fibrosis is an essential component of tissue repair that follows tissue injury and is usually associated with inflammation. Progressive fibrosis reflects a pathologic state and results in scarring and impairment of organ function. Myocardial fibrosis is a hallmark in end‐stage DCM, starting with potentially reversible diffuse reactive fibrosis finally transitioning to irreversible focal scarring fibrosis.^97^ The main pathways and biological entities involved in the development of myocardial fibrosis are the mineralocorticoid and transforming growth factor‐β pathways, and nonstructural matrix proteins and micro‐RNAs.^98^ To date, the impact of gene mutations and additional environmentally acquired factors on the extent of myocardial fibrosis is largely unknown. Some insights have been gained by examining subsets of patients with DCM such as those with *LMNA* mutations where premature scarring fibrosis is associated with increased risk of arrhythmias.^45^ In this regard, targeting fibrosis can be roughly divided into 2 categories.

The first category is delaying or preventing the onset of myocardial fibrosis, with early detection of potentially treatable causes to prevent further progression toward irreversible fibrosis in DCM patients or those at risk of developing DCM. The second category is regression or stabilization of established myocardial fibrosis through targeting of key molecular processes. Although these key molecular processes can be targeted in several ways, most strategies do not specifically target fibrosis.

Mineralocorticoid receptor antagonists, already part of standard HF therapy, may be interesting antifibrotic drugs because they act on extracellular matrix remodeling, decrease collagen biosynthesis biomarkers, and improve outcomes in patients with HF and DCM.^44^, ^99^ In DCM patients, myocardial fibrosis on a histologic level can be reduced using mineralocorticoid receptor antagonists accompanied by a decrease in collagen biosynthesis biomarkers.^44^ Moreover, myocardial gal‐3 (galectin‐3) plasma concentrations are increased by aldosterone and angiotensin II early in the fibrotic cascade and can be reduced by mineralocorticoid receptor antagonists, consistent with an effect of these agents on tissue pathology.^99^ Oher potential therapeutic targets include transforming growth factor‐β, interleukins, and wnt signaling, because they can transform quiescent fibroblasts into active collagen‐producing myofibroblasts.^100^ Although the molecular transforming growth factor‐β pathway is important in virtually all types of fibrosis, its pleiotropic effects make it an unattractive target. Cardiotrophin‐1, a member of the IL‐6 superfamily, is a profibrotic factor and is increased in the myocardium of patients with HF of different causes, including DCM.^101^ Finally, inhibition of wnt signaling reduced fibrosis and cardiac recovery in mice.^102^ So far, pharmacological studies are performed only in gal‐3 and wnt signaling. Gal‐3 inhibitors include carbohydrates such as modified citrus pectin, which is being used in an ongoing trial including patients with high blood pressure to reduce cardiac fibrosis (NCT01960946). Interestingly, a phase 1 trial using a monoclonal antibody called vantictumab, which inhibits downstream signaling of wnt, was completed in December 2017, results of which are still being awaited (NCT01973309). Thus, targeting the angiotensin II‐galectin‐3‐IL‐6 axis using mineralocorticoid receptor antagonists or Gal‐3 inhibitors or inhibiting wnt signaling pathway by vantictumab could specifically reduce cardiac fibrosis.

Matricellular proteins have a potential in antifibrotic therapies, because they can degrade collagen and modulate the function of cardiac fibroblasts, inflammatory cells, and cardiomyocytes. Recently, the first proof of principle for a therapeutic potential has evolved for osteoglycin. Absence of osteoglycin in mice reduced collagen cross‐linking in the infarct scar after myocardial infarction, thereby increasing cardiac rupture and dilatation. Osteoglycin administration through gene therapy enhanced collagen maturation and thereby prevented LV dilatation and dysfunction after myocardial infarction.^103^ To date, no human trials exist.

Various small noncoding RNAs (miRs), posttranscriptional regulators of gene expression, are involved in cardiac fibrosis. Myocardial fibrosis in patients with diverse cardiac disease is accompanied by increased expression of profibrotic miRs including miR‐21, miR‐208a, and miR‐499‐5p, and decreased expression of antifibrotic miRs such as miR‐29, miR 19b, miR‐1, miR‐133a, and miR‐122.^98^ More importantly, miRNAs can be easily manipulated and provide a novel class of antifibrotic agents. Although no human trials including HF patients receiving miRs are conducted yet, the antifibrotic miR‐122 reached phase II trial for treating hepatitis.^104^ Thus, the advancement of miR‐based therapeutics into the clinical testing of HF is on the horizon.

Besides quantity is the quality of myocardial fibrosis, in terms of collagen cross‐linking, which is an equally important feature.^97^ Excessively cross‐linked collagen is difficult to degrade and critically affects extracellular matrix turnover. Syndecan‐4‐osteopontin‐Lysyl Oxidase‐Like axis is important in the formation of insoluble cross‐linked collagen, and therapeutic strategies that target such pathways could also ameliorate the effects of myocardial fibrosis.^105^ A monoclonal antibody against Lysyl Oxidase‐Like 2, called simtuzumab, is currently being tested in a phase IIb trial in subjects with compensated cirrhosis secondary to nonalcoholic steatohepatitis (NCT01672879). So far, no clinical trials in cardiac diseases have been initiated.

Interestingly, in the field of idiopathic pulmonary fibrosis, 2 antifibrotic medications, nintedanib and pirfenidone, have been shown to be safe and effective in the treatment of idiopathic pulmonary fibrosis and are currently recommended for use in these patients.^106^ Nintedanib is a tyrosine kinase inhibitor that targets growth factor pathways and pirfenidone has a number of anti‐inflammatory and antifibrotic effects, including inhibition of collagen synthesis, downregulation of transforming growth factor‐β and tumor necrosis factor‐α, and a reduction in fibroblast proliferation.^107^ Currently, a phase II trial is being conducted to evaluate the efficacy and safety of pirfenidone in patients with HF and preserved ejection fraction (NCT02932566).

## Clinical Implications and Future Perspectives

The greatest pitfall of evidence‐based cause‐directed therapy is the small number of patients included in studies (<100) (Table 2). This is mainly because of the heterogeneous nature of DCM and incomplete characterization of cause, which limits the numbers of specific subgroups of patients. Often echocardiographic analysis is available without CMR, and endomyocardial biopsy is not standard in all countries/practices. Although the quality of most studies is good, the majority remain single‐center experience. Therefore, clinical implications are limited. The only guideline‐changing therapies over the past few years were tested in general HF, of which DCM is often a small subset. This retains the current dogma of nonpersonalized treatment.

**Figure 3:**
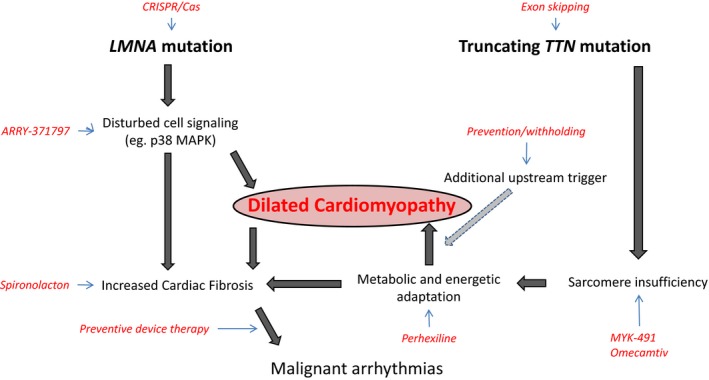
Two examples of genetic mutations leading to dilated cardiomyopathy and their potential treatment targets interfering with disease progression at different levels.

Understanding the molecular consequences of genetic DCM will provide novel and specific treatment targets. *LMNA* DCM is a good example, as the altered intracellular signaling is well studied. Therefore, multiple therapies can interfere with the disease course, upstream and downstream (Figure 3). With the high prevalence of *TTN*‐associated DCM, its molecular and phenotypical signature is becoming more clear. This will also provide therapeutic options for these specific patients. Although we have a broad selection of therapies, we now have to fine‐tune the therapy based on upstream triggers and their downstream active processes (Figure 3).

Genetic diagnostics adds a different dimension to the spectrum. The increase in genotype–phenotype knowledge will greatly benefit prevention and a personalized approach. Early detection and preventive medicine will be an important focus of future research. Clinical and genetic screening of DCM relatives is a perfect example for identifying carriers of a pathogenic mutation who are at risk for developing the phenotype. Herein, global longitudinal strain may provide a useful tool to detect subclinical disease in “healthy” DCM relatives and moreover carries prognostic relevance. However, there is great clinical variability and incomplete penetrance within families because of factors we do not understand yet. Moreover, whether prophylactic treatment is beneficial and able to prevent disease onset is unknown. This is an important area of research because it will greatly benefit patient care and provide reassurance among genetic DCM families.

Another important clinical issue is the safety of medication withdrawal in currently asymptomatic DCM patients with complete cardiac recovery. Notably, DCM patients are relatively young and reluctant to take lifelong medication without continued beneficial effects. A recent open‐label, randomized, pilot trial suggested that ≈40% of patients with recovered DCM will have a relapse within 6 months after phased medication discontinuation.^108^ However, this also showed that 60% of DCM patients did not have a relapse over that time period. Future research should focus on identifying subgroups of DCM patients in whom pharmacological treatment for HF can be safely withdrawn. One such possible marker for identifying subgroups is global longitudinal strain analysis on echocardiography. A recent study showed that 79% of recovered HF patients still had an abnormal global longitudinal strain, despite normalization of geometry and/or LVEF.^109^ Furthermore, the abnormal global longitudinal strain is a predictor for worse clinical outcome in general recovered HF patients.

The ultimate goal is to link the key disease‐driving mechanisms in DCM patients with specific therapies. These targeted therapies will be most beneficial in those patients who do not improve sufficiently using the established guideline‐directed medical therapies.^17^ This should set the stage for further in‐depth pheno‐ and genotyping using the proposed diagnostic DCM workup (Figure 2). As such, standardized classification of DCM subclasses according to the upstream cause and downstream molecular processes at the time of clinical evaluation is crucial. Herein, the Morphology, Organ Involvement, Genetic, Etiology, Stage of disease classification can provide the first steps toward standardized classification of these subclasses who might benefit most from targeted therapy (eg, immunosuppressive, antifibrotic therapies). Once global standardized classification and subsequent stratification validates the proposed up‐ and downstream subclasses, multicenter trials using targeted therapies adjunctive to established guideline‐directed medical therapy can be initiated to prove their incremental value. In the future, recruitment should start in global expert centers with the availability of advanced imaging technologies (eg, CMR), genetic evaluation, and preferably myocardial biopsy. Treatment efficacy should be monitored using established (natriuretic peptides, troponin) and relevant circulating biomarkers for the specific therapy (eg, inflammatory markers for immunosuppression, fibrosis markers for antifibrotic therapy). In addition, assessment of short‐term (6–12 months) functional improvement such as HF questionnaires, exercise tolerance, and cardiac function together with “hard” end points including HF hospitalization, life‐threatening arrhythmias, implantation of ventricular assist device, heart transplantation, and death are crucial to evaluate long‐term beneficial effects of the targeted therapy.

## Conclusions

DCM constitutes a broad clinical phenotype that arises from a final common response to a number of genetic and environmentally acquired insults. Detecting the underlying dominant cause and the ongoing downstream pathophysiological processes is the key to initiate targeted treatment strategies. Several small targeted trials have been conducted, but as of now the era has arrived to conduct large, multicenter trials that result in breakthroughs in targeted treatment that improves prognosis in these patients.

## Sources of Funding

This work was supported by the European Union Commission Seventh Framework Programme under grant agreement No. 305507 (HOMAGE). Kootstra Talent Fellowship of the Maastricht UMC+ to Dr Hazebroek. Dr Ware received funding from Wellcome Trust (107469/Z/15/Z). Dr Prasad has received funding from British Heart Foundation, Rosetrees, and Alexander Jansons Foundation. We also acknowledge the support from the Netherlands Cardiovascular Research Initiative, an initiative with support of the Dutch Heart Foundation, CVON2011‐ARENA, CVON2016‐Early HFPEF, and CVON 2017‐ShePREDICTS. This research is cofinanced as a PPP Allowance Research and Innovation by the Ministry of Economic Affairs within Top Sector Life Sciences & Health.

## Disclosures

Dr Ware did consultancy work for MyoKardia. The remaining authors have no disclosures to report.
